# Fast Switchable Dual-Model Grating by Using Polymer-Stabilized Sphere Phase Liquid Crystal

**DOI:** 10.3390/polym10080884

**Published:** 2018-08-08

**Authors:** Xuan Li, Xiaowei Du, Peiyun Guo, Jiliang Zhu, Wenjiang Ye, Qin Xu, Yubao Sun

**Affiliations:** Department of Applied Physics, Hebei University of Technology, Tianjin 300401, China; lixuan576675@163.com (X.L.); xiaoweidu5200@163.com (X.D.); peiyun_guo@163.com (P.G.); shengze_ye@163.com (W.Y.); xuqinzi@126.com (Q.X.); sun_yubao@163.com (Y.S.)

**Keywords:** liquid crystals, LC polymer, diffraction grating, fast response time

## Abstract

We demonstrated a fast switchable dual-model grating based on a polymer-stabilized sphere phase liquid crystal. To form binary periodicity layers, the polymer-stabilized sphere phase liquid crystal precursor was sequence ultraviolet cured at an isotropic and sphere phase. This grating jointly modulated both the phase and the amplitude, had six times the diffraction efficiency of that fabricated with polymer-stabilized blue phase liquid crystal. Moreover, the dual-model tunable grating shown polarization-independent and submillisecond response time, which may hold a great potential application in diffractive optics.

## 1. Introduction

Liquid crystal (LC) grating has been widely used in manufacture of three-dimensional (3D) displays [1], optical communication [2], and beam steering [3,4] because of its interesting features, such as electrically tunable diffraction efficiency, low energy consumption, high resolution, and so on. According to the modulation mode of incident light, the diffraction grating has two categories: the amplitude grating and the phase grating. LCs with electrically controlled birefringence have been widely used to fabricate the phase gratings [5]. Several methods adopted to achieve the periodic change of refractive index in the LC phase grating are as follows: periodic electric field distribution induced by striped or patterned electrode [6,7], patterned alignment layer [8], and grating-like periodic LC layer [9,10,11]. Among them, photo-polymerisation is consider as one of good ways to fabricate the grating-like periodic LC layer. By using two-step ultraviolet (UV) radiation with a photomask, Lin et al. proposed a polymer-stabilized (PS) blue phase (BP) LC (PS-BPLC) phase grating with 0.8% of diffraction efficiency [9]. Nejmettin et al. demonstrated an LC phase grating by the photo-polymerisation-induced phase separation. Yuan et al. used a novel one-step holographic fabrication to make a tunable PS-BPLC phase grating of ~4 μm grating constant. In addition, common LC materials have been investigated to form LC phase gratings, including nematic [12,13], cholesteric [14,15]. smectic [16], and blue phases [17,18]. Compared to a pure phase grating, two-model LC grating with phase and amplitude switched simultaneously is much less studied [19,20]. 

Recently, a self-assemble sphere phase (SP) LC has attracted researchers’ intensive attention for its fantastic features and potential applications in displays, 3D tunable photonic crystals [21,22,23,24,25,26], and phase modulators [27]. The SP consists of 3D twist spheres (3-DTSs) and unstable disclinations among them. By introducing the polymer network into the disclinations, the temperature range of SP can be achieved over 80 °C covering the room temperature. The PS sphere phase LC (PS-SPLC) can be switched between haze and transparent states in submillisecond under an electric field of 4.4 V/μm. Due to the combination of scattering and interference effects, the PS-SPLC can been used as a chaotic amplifying medium of the laser with a low threshold energy. The central wavelength of PS-SPLC laser can be tunable in 40 nm by electric field [28]. Recently, our group proposed an extended anomalous diffraction approach model to explain the electro-optical properties of PS-SPLC devices [29]. Because of its perfect electro-optical properties, the PS-SPLC is promising for making LC grating.

In this work, an electrically tunable two-model grating is proposed and demonstrated based on a PS-SPLC. Experimental results indicated that this grating jointly modulates both the phase and the amplitude of incident light, the diffraction efficiency of first order reaches 4.37%, which is higher than that of the homotype LC grating. In addition, this LC grating also shows the merits of polarization-independence, submillisecond response time, and simple manufacture process.

## 2. Materials and Methods

### 2.1. Method of Fabricating Liquid Crystal Gratings

**Figure 1:**
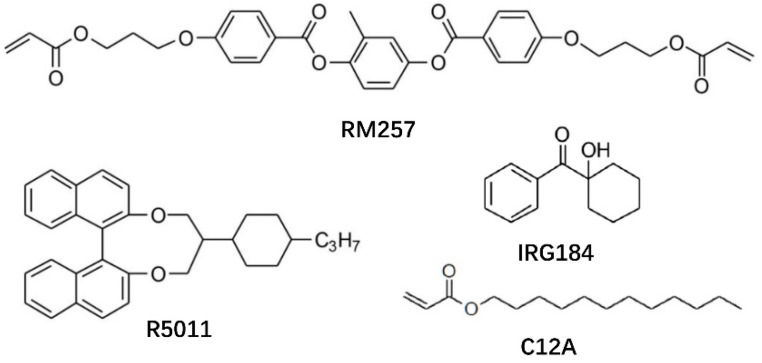
Chemical structures of chiral dopant, monomers, and photoinitiator.

The SPLC mixture used in this work consists of 84.3 wt. % of a nematic LC host (Δn = 0.15, Δε = 29, HCCH) and 5 wt. % of a chiral dopant R5011 (HCCH). The phase sequence of the mixture during cooling was isotropic (Iso)-67.7 °C-SP-63.1 °C-BP-53.6 °C-chiral nematic phase (N*). To stabilize the disclinations among the 3-DTSs, two photo-curable monomers (5 wt. % C12A and 5 wt. % RM257, HCCH) and a photoinitiator (0.7 wt. % IRG 184, HCCH) were doped into the SPLC mixture. Then, the homogeneous PS-SPLC precursor was sandwiched between two thin indium-tin-oxide (ITO) glass substrates without alignment layer at high temperature so that the LCs were in Iso. The mixture showed the following phase sequence during the cooling at a rate of 0.1° Cmin^−1^: Iso-53.8 °C-SP-45 °C-BP-35.7 °C-N*. Chemical structures of chiral dopant, monomers, and photoinitiator were given in Figure 1. All materials were used as received.

**Figure 2:**
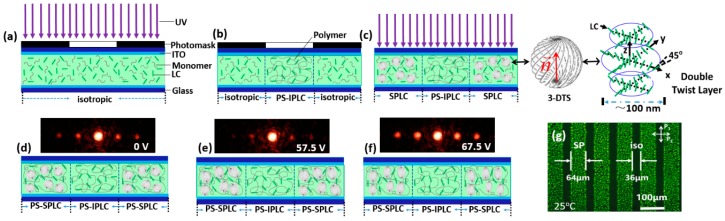
(**a**–**c**) Schematic diagram of the polymer-stabilized sphere phase liquid crystal (PS-SPLC) grating fabrication process. Diffraction pattern and possible schematic diagram of liquid crystalline molecular orientation of the PS-SPLC grating at (**d**) V = 0 V, (**e**) 57.5 V, and (**f**) 67.5 V. (**g**) the polarizing micrographs of PS-SPLC grating in reflection mode at the turn-off state.

To fabricate the PS-SPLC grating, a photomask, a grating of 100 μm grating constant, was placed on the top glass substrate of the sample at 63 °C, as shown in Figure 2a. After the sample was cooled down to 60 °C at a rate of 0.1 °C/min, the sample was exposed under collimated UV light (365 nm) with an intensity of 3 mW/cm^2^ for 7 min. In bright zones, the sample was stabilized in an isotropic and the dark zones blocked by the photomask was not exposed, as shown in Figure 1b. After the first exposure, the sample was cooled to the SP at 54 °C, then was exposed under the collimated UV light for 5 min to stabilize the uncured zones (Figure 2c). Because of the monomer concentration difference between the dark zones and the bright zones, the monomers may diffuse from the dark zones to the bright zones during the first exposure, resulting in the temperature increment of the SP. The LC grating under alternate PS-SPLC and PS isotropic phase (IP) LC was thus achieved (Figure 2d). As illustrated in Figure 2g, the PS-SPLC zones is slightly larger than that of the PS-IPLC. This phenomenon might be attributed to two factors: (1) a portion of PS-SPLC precursors dispersed from the dark zones to the exposed zones during the first exposure process, and (2) the PS-IPLC at the age of the photomask was unstable due to the incomplete polymerization of monomer. The width of the isotropic zones can be changed by appropriately adjusting the time of the first exposure. Figure 2d–f shows the diffraction pattern of the LC grating at different applied voltages.

### 2.2. Characterization

**Figure 3:**
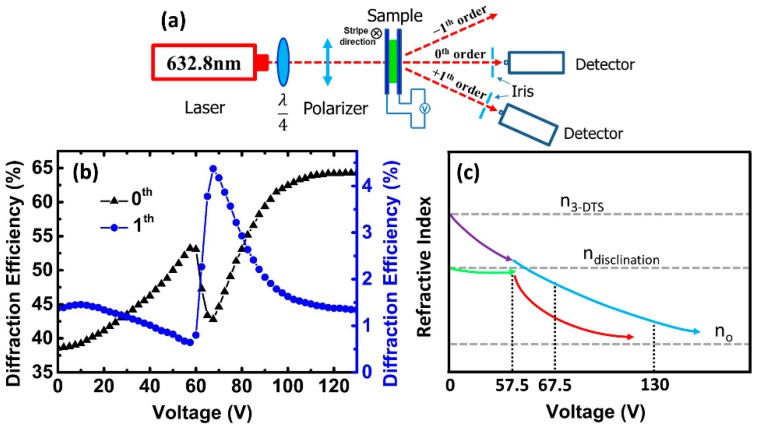
(**a**) Experiment setup for measuring the diffraction efficiency of the PS-SPLC grating. (**b**) Voltage dependent diffraction efficiencies of the zeroth and first orders. (**c**) Schematic diagram of the refractive index mismatch between the 3D twist spheres (3-DTSs) (*n*_3-*DTS*_) and the disclinations (*n*_disclination_).

Figure 3a shows the experimental setup used to measure the diffraction efficiency at room temperature (25 °C). A 632.8 nm linear polarized He-Ne laser was converted into a circularly polarized light after passing through a *λ*/4 plate. The polarization direction of the incident light can be kited by a polarizer behind the *λ*/4 plate. The irises behind the sample were used to restrict the scattered light into the detector and select diffraction orders. The intensity of the diffraction orders was detected by detectors with ~50 cm distance from the sample.

### 2.3. Fundamentals of LC Gratings

The diffraction efficiency of this LC grating mainly depends on the refractive index difference between the PS-SPLC (*n_PS-SPLC_*) and the PS-IPLC (*n_PS-IPLC_*) and the effective amplitude of the incident light. In this LC grating, the phase difference Δ*φ* (*E*) between the PS-SPLC zone and the PS-IPLC zone can be given as Equation (1):

(1) $$\Delta \varphi \left(E\right)=\frac{2\pi }{\lambda }\left[n_{PS-SPLC}\left(E\right)-n_{PS-IPLC}\left(E\right)\right]d$$

where *d* is the cell gap and *λ* is the wavelength of the incident light. After, a Fourier transform of complex amplitude distribution of grating emergent plane was conducted to calculate the diffracted efficiency of each order. The diffraction efficiency on zeroth and first orders can be expressed as Equations (2) and (3) [30,31]:

(2) $$\eta_{0}=T_{1}\left(E\right)\rho^{2}+T_{2}\left(E\right){\left(1-\rho \right)}^{2}+2\sqrt{T_{1}\left(E\right)T_{2}\left(E\right)}\times \rho \left(1-\rho \right)\cos\left(\Delta \varphi \right)$$

(3) $$\eta_{1}=\frac{1}{2\pi^{2}}\left[T_{1}\left(E\right)+T_{2}\left(E\right)-2\sqrt{T_{1}\left(E\right)T_{2}\left(E\right)}\cos\left(\Delta \varphi \right)\right]\left[1-\cos\left(2\pi \rho \right)\right],$$

where *T*_1_(*E*) and *T*_2_(*E*) are the effective transmittances of PS-SPLC and PS-IPLC, respectively. The duty cycle of PS-SPLC in this LC grating is *ρ* ≈ 0.64, which is defined as a ratio between the width of PS-SPLC zone and the grating constant.

## 3. Results

### 3.1. Diffraction Efficiency of LC Gratings

At the turn-off state, the clear diffraction effect was observed because of the refractive index difference between the PS-SPLC and the PS-IPLC, as shown in Figure 2d. As a square-wave signal of 1 kHz frequency was applied on the LC grating, the diffraction efficiency on the first order decreased with the applied voltage increased to 57.5 V. Then, the diffraction efficiency reached to a highest one at 67.5 V (Figure 2f). Figure 3b depicts the voltage dependent diffraction efficiency of the LC grating on zeroth and first orders. The diffraction efficiency was defined as a ratio between the intensity of mth diffraction order and the total intensity of the incident light. At the turn-off state, the diffraction efficiency of the grating on zeroth and first orders were 38.6% and 1.38%. The lower diffraction efficiency originates from the light scattering in PS-SPLC induced by the refractive index mismatch between the 3-DTSs and the disclinations among them. When the applied voltage increased, the light scattering in PS-SPLC decreased, which, in turn, increased the amplitude (effective incident light) of LC grating, making the diffractive efficiency of zeroth order increased to 53.2% from 38.5%. Therefore, the amplitude of LC grating could be adjusted by the light scattering. However, the phase change between the PS-SPLC and the PS-IPLC decreased as the applied voltage increased, resulting in the diffractive efficiency on first order decreased. At 57.5 V, the diffractive efficiency of the LC grating on first order was ~0.64%. When the applied voltage increased from 57.5 V to 67.5 V, the diffraction efficiency of the LC grating on zeroth order decreased from 53.2% to 42.8% and that on first order increased from 0.64% to 4.37%, which is about six times of that fabricated with the same method in reference 9. This special phenomenon was attributed to the light scattering of PS-SPLC, which decreased the amplitude of the LC grating, and the phase change of the LC grating. As the applied voltage increased, the scattering of PS-SPLC become stronger gradually due to the refractive index mismatch between 3-DTSs and the coexistence of disclinations at an isotropic state stabilized with polymer network, resulting in the diffractive efficiency decrement of the zeroth order. Meanwhile, the refractive index difference between PS-SPLC and PS-IPLC increased, which made the diffractive efficiency of first order increased. Upon further increasing the applied voltage, the diffractive effect become weaker owing to that the refractive index of PS-SPLC and PS-IPLC tends to the ordinary refractive index (*n_o_*) of the nematic LC host.

Figure 3c shows the schematic diagram of the refractive index mismatch between the 3-DTS (*n*_3-*DTS*_) and the disclination (*n*_disclination_). At the voltage-off state, *n*_3-*DTS*_ > *n*_disclination_ > *n_o_*. The overall refractive index of the PS-SPLC (*n_PS-SPLC_*) depended on the *n*_3-*DTS*_ and the *n*_disclination_. The driving voltage of the LC molecules in the disclinations was higher than that in the 3-DTS owing to the strong anchoring energy of the polymers. Because of the anchoring energy of the polymers, the LC molecules in 3-DTS turned to align with the applied electric field prior to that in the disclinations. As a result, before the voltage reached 57.5 V, *n*_3-*DTS*_ tends to *n*_o_ early than *n*_disclination_, as shown in Figure 3c (purple line and green line). The refractive index difference between the *n*_3-*DTS*_ and the *n*_disclination_ was reduced, resulting in a transition from scattering to transmission. After, the LC molecules in the disclinations rapidly turn to align with the applied electric field (the *n*_disclination_ rapidly transitions to *n_o_*) when the voltage is larger than 57.5 V. The refractive index difference between the *n*_3-*DTS*_ and the *n*_disclination_ rapidly increases, so that the scattering increases, as show in Figure 3c (red line). Finally, both the molecules in the 3-DTS and the disclination were aligned with the applied electric field, resulting in that *n*_3-*DTS*_ and *n*_disclination_ both reach to *n_o_* and the transmittance reaches to a maximum value at 130 V (Figure 3c, blue line).

### 3.2. Diffraction Efficiency of LC Gratings

**Figure 4:**
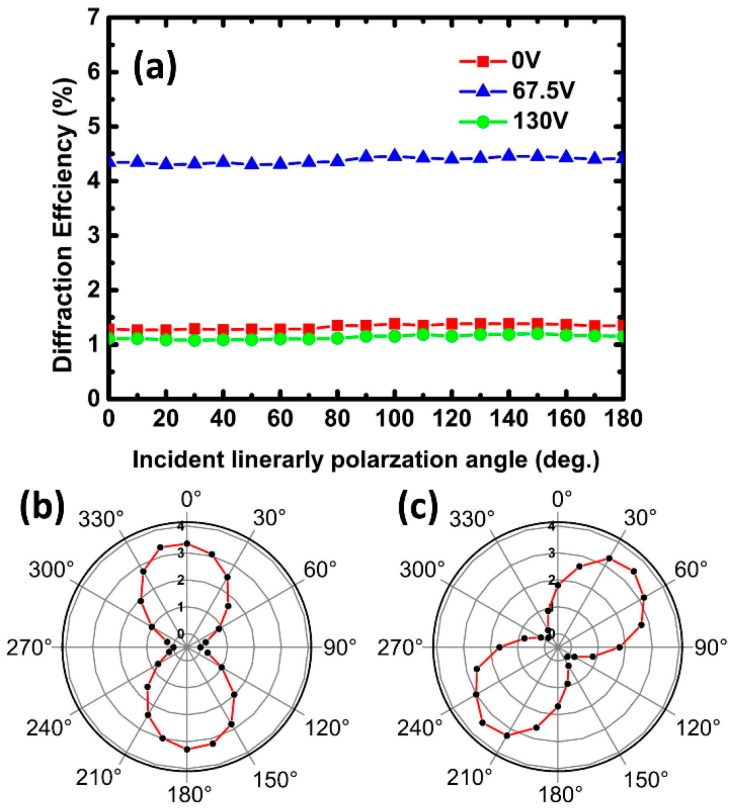
(**a**) The first order diffraction efficiency with different incident light polarization angles at applied voltages of V = 0 V, 67.5 V, and 130 V. (**b**,**c**) Polarization of first order diffracted beam with 0° and 45° polarized incident light at 67.5 V, respectively.

The polarization property of this LC grating was investigated by changing the incident light. Figure 4a shows the first order diffraction efficiency versus polarization angle of incident light at applied voltages of 0 V, 67.5 V, and 130 V, respectively. The results show that the diffraction efficiency on first order does not vary with the polarization angle. The positive dielectric anisotropy of SPLC makes the LC director tend to align with the electric field, as a result, the direction of the electric field vector of incident light is perpendicular to the optical axis of the LC. Hence, the PS-SPLC grating is polarization-independence.

Figure 4b,c show the polarization of the first order diffracted beam with 0° and 45° polarized incident beams at 67.5 V, respectively. Measurement results indicate that the polarization direction of the diffraction spot is the same as the incident light. Therefore, the applied voltage only changes the diffraction efficiency of PS-SPLC grating, and has no effect on the polarization of incident light. The PS-SPLC grating is polarization-independent.

### 3.3. Response Time of LC Gratings

**Figure 5:**
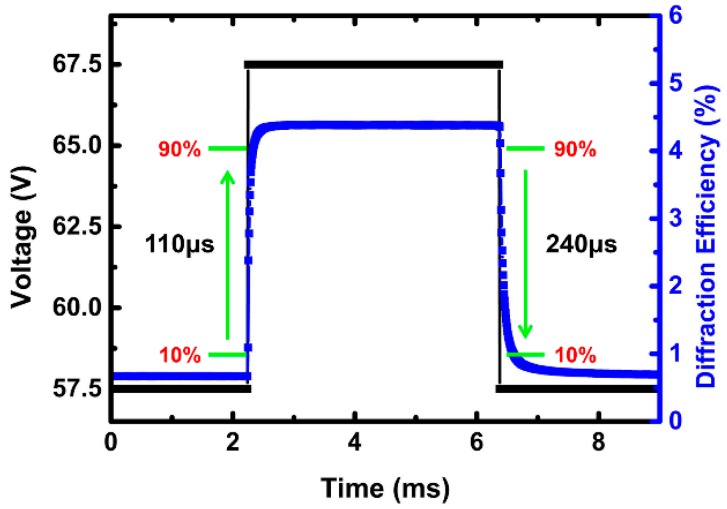
Plots the electro-optical response of rise time and decay time of the liquid crystal (LC) grating on first order.

The response time is an important parameter of a LC grating. The rise and decay times are determined as the change of the diffraction efficiency from 10 to 90% and 90 to 10%, respectively. As show in Figure 5, the rise time is 110 μs and the decay time is 240 μs at the room temperature. The total response time of this grating is 350 μs, which is one tenth of the nematic LC gratings.

### 3.4. Discussion

**Figure 6:**
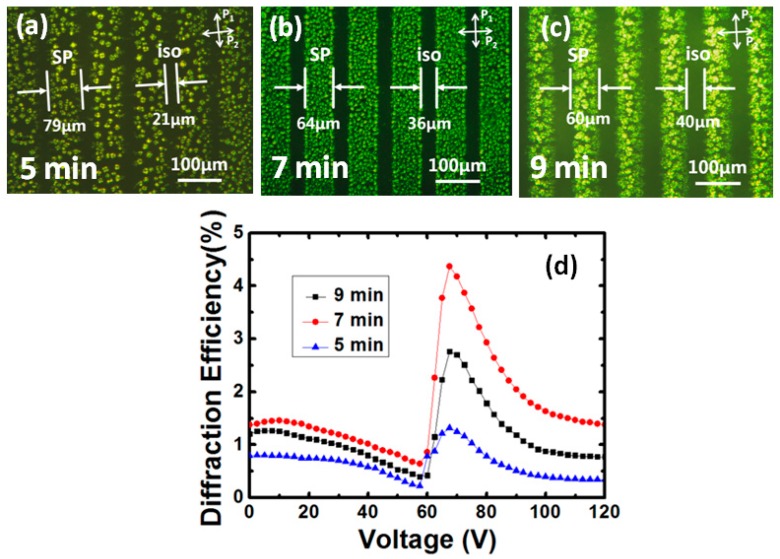
Monomer diffusion effect on the properties of the LC grating. (**a**–**c**) The polarizing micrographs of PS-SPLC grating with different diffusion time and (**d**) the diffraction efficiency on first order with different diffusion time.

As aforementioned, the monomer diffusion from the dark zones to the bright zones occurred during the first exposure, the concentration of the polymer fibrils in the PS-IPLC zones is higher than that in the PS-SPLC zones. Therefore, optimizing the time of the first-step illumination has been done to investigate the ratio of polymer network effect on the properties of this grating, as shown in Figure 6. The *ρ* decreases as the expose time increases from 5 to 9 min. The insufficient polymerization with less expose time makes the isotropic zones unstable, the SP appeared in the isotropic zones when the sample was cooled to the SP before the second-step illumination (Figure 6a), resulting in a lower diffraction efficiency (Figure 6d). The longer expose time is, much more monomers diffused from the dark zones to the bright zones, resulting in a higher phase transition temperature from an isotropic to SP and a lower diffractive efficiency, as shown in Figure 6c. If the exposure time was too long, the N* instead of the SP appeared after the first-step illumination. The experimental results also shown that the monomer diffusion has less effect on the driving voltage and response time because they are effective values depended on the chiral dopant and the total ratio of the polymer fibrils between the PS-IPLC zones and the PS-SPLC zones.

## 4. Conclusions

In conclusion, we have demonstrated a dual-model LC grating with PS-SPLC. The diffraction efficiency can be modulated by controlling the external voltage to change the phase and amplitude. The diffraction efficiency of this LC grating on the first order is 1.38% at voltage-off state. When the voltage was 67.5 V, the diffraction efficiency achieved a maximum value, 4.37%, which was six times of that fabricated with PS-BPLC. Moreover, this device also possesses polarization-independent and sub-millisecond response time, which holds a great potential for diffractive optics. We provided a new way to research the LC grating.
